# MATE2 Expression Is Associated with Cancer Cell Response to Metformin

**DOI:** 10.1371/journal.pone.0165214

**Published:** 2016-12-13

**Authors:** Sanjana Chowdhury, Eric Yung, Melania Pintilie, Hala Muaddi, Selim Chaib, ManTek Yeung, Manlio Fusciello, Jenna Sykes, Bethany Pitcher, Anna Hagenkort, Trevor McKee, Ravi Vellanki, Eric Chen, Robert G. Bristow, Bradly G. Wouters, Marianne Koritzinsky

**Affiliations:** 1 Princess Margaret Cancer Centre, University Health Network, Toronto, Canada; 2 Institute of Medical Science, University of Toronto, Toronto, Canada; 3 University of Maastricht, Maastricht, The Netherlands; 4 Department of Medical Biophysics, University of Toronto, Toronto, Canada; 5 Department of Radiation Oncology, University of Toronto, Toronto, Canada; National Cancer Institute, UNITED STATES

## Abstract

**Background:**

There is great interest in repurposing the commonly prescribed anti-diabetic drug metformin for cancer therapy. Intracellular uptake and retention of metformin is affected by the expression of organic cation transporters (OCT) 1–3 and by multidrug and toxic compound extrusion (MATE) 1–2. Inside cells, metformin inhibits mitochondrial function, which leads to reduced oxygen consumption and inhibition of proliferation. Reduced oxygen consumption can lead to improved tumor oxygenation and radiation response.

**Purpose:**

Here we sought to determine if there is an association between the effects of metformin on inhibiting oxygen consumption, proliferation and expression of OCTs and MATEs in a panel of 19 cancer cell lines.

**Results:**

There was relatively large variability in the anti-proliferative response of different cell lines to metformin, with a subset of cell lines being very resistant. In contrast, all cell lines demonstrated sensitivity to the inhibition of oxygen consumption by metformin, with relatively small variation. The expression of OCT1 correlated with expression of both OCT2 and OCT3. OCT1 and OCT2 were relatively uniformly expressed, whereas expression of OCT3, MATE1 and MATE2 showed substantial variation across lines. There were statistically significant associations between resistance to inhibition of proliferation and MATE2 expression, as well as between sensitivity to inhibition of oxygen consumption and OCT3 expression. One cell line (LNCaP) with high OCT3 and low MATE2 expression in concert, had substantially higher intracellular metformin concentration than other cell lines, and was exquisitely sensitive to both anti-proliferative and anti-respiratory effects. In all other cell lines, the concentration of metformin required to inhibit oxygen consumption acutely *in vitro* was substantially higher than that achieved in the plasma of diabetic patients. However, administering anti-diabetic doses of metformin to tumor-bearing mice resulted in intratumoral accumulation of metformin and reduced hypoxic tumor fractions.

**Conclusions:**

All cancer cells are susceptible to inhibition of oxygen consumption by metformin, which results in reduced hypoxic tumor fractions beneficial for the response to radiotherapy. High MATE2 expression may result in resistance to the anti-proliferative effect of metformin and should be considered as a negative predictive biomarker in clinical trials.

## Introduction

Metformin is a biguanide commonly prescribed as first line oral therapy in the treatment of type 2 diabetes [1]. There is great interest in repositioning metformin for use in cancer, spurred by data indicating that metformin use in diabetic patients is associated with decreased incidence of neoplastic disease [1–5]. Metformin use has also been associated with improved outcome following cancer therapy in retrospective studies [6–13]. Interestingly, several small early-phase prospective studies have recently shown that metformin treatment results in reduced tumor proliferation when given to non-diabetic cancer patients [14–17], suggesting that metformin may have a causative role in improving outcome.

The cellular targets of metformin are mitochondrial glycerophosphate dehydrogenase (mGPD) and respiratory complex I in the mitochondrial electron transport chain (ETC) [18–20]. The primary effect of metformin is therefore reduction of mitochondrial oxygen consumption and activity. In response to this, cells mount a multifaceted adaptation response governed by activation of AMPK and p53 that ultimately leads to reduced proliferation [21, 22]. Importantly, both the primary effect of reducing oxygen consumption as well as the secondary effect of reducing cell proliferation may be of benefit to cancer patients. High oxygen consumption in cancer cells proximal to tumor vessels results in oxygen gradients and hypoxia in more distal tumor volumes [10, 23–25]. Clinically, tumor hypoxia is a negative prognostic factor due to the increased aggressiveness of hypoxic cancer cells, combined with their decreased response to radio- and chemotherapy [26–28]. Using experimental animal models, we have recently demonstrated that the inhibition of mitochondrial respiration by metformin improves oxygen distribution in tumors and potentiates radiation response [10].

With metformin entering prospective clinical trials for use in combined modality cancer treatment, identification of appropriate biomarkers is warranted in order to stratify the patient population. One important parameter to consider, which would be expected to be essential for both direct modulation of hypoxia and proliferation, is the intracellular concentration that can be achieved. Metformin depends upon the organic cation transporters OCT1, OCT2, OCT3 (*SLC22A1*, *SLC22A2*, *SLC22A3*) for its cellular uptake, and multidrug and toxin extrusion transporters MATE1 and MATE2 (*SLC47A1* and *SLC47A2*) for its excretion [29]. OCTs are a family of poly-specific bidirectional uniporters while MATEs are bidirectional H^+^/organic cation antiporters [30, 31]. OCT1 is most abundantly expressed in hepatocytes [30], which is thought to underlie the primary effect of metformin in diabetic patients, namely reducing liver gluconeogenesis [32]. OCT2 and MATE2 are primarily expressed in renal tubule cells that are responsible for the renal excretion of metformin [29, 31]. OCT3 is highly expressed in muscle and adipose tissue [33]. Single nucleotide polymorphisms affecting the function of OCT1, OCT2, MATE1, and MATE2 have been linked to altered metformin disposition, pharmacokinetics, response and side-effects in diabetic patients [34–49]. The expression of OCTs and MATEs in cancerous cells or tissues however, has not been extensively studied. Ectopic expression of OCT3 has been shown to increase uptake of and response to metformin in breast cancer cells [50], but whether basal OCT1-3 or MATE1-2 expression predict response remains unknown.

Here, we sought to determine the potential roles of OCT and MATE expression as predictive biomarkers of sensitivity to the anti-proliferative and the respiratory-inhibitory effects of metformin *in vitro*. We also investigated whether the inhibition of respiration observed *in vitro* could translate to reduced tumor hypoxia *in vivo* when metformin was administered to tumor-bearing mice at anti-diabetic doses.

## Materials & Methods

### Cell lines and culture

HCT116 (colorectal carcinoma), LNCaP (prostate carcinoma), HCC827 (non- small cell lung adenocarcinoma) and BxPC3 (pancreas adenocarcinoma) cells were cultured in RPMI 1640 media. HepG2 (hepatocellular carcinoma) and RCC4 (renal carcinoma) cells were cultured in DMEM media. HeLa and ME-180 cervical carcinoma cell lines were cultured in alpha-MEM media. A549 lung carcinoma cells were cultured in DMEM:F12 media. T98G glioblastoma multiforme cells were cultured in EMEM media. The head-and-neck cancer cell lines FaDu, SCC-16A, SCC-16B, SCC-19A, SCC-19B, SCC-74A, and SCC-74B were all cultured in MEM-F15 media. All these cell lines were cultured as adherent monolayers in exponential growth phase in the indicated media (Gibco) supplemented with 10% fetal bovine serum (Gibco). U937 (histiocytic lymphoma of myeloid lineage) and POP-092S (colon adenocarcinoma) cells were cultured in suspension in RPMI 1640 or growth media to enrich for stem cells [51] respectively. The patient-derived head-and-neck cancer cell lines (SCC-16A/B, SCC-19A/b, SCC-74A/B) were generously provided by Dr. R. Grenman from Turku University Hospital (Finland), POP-092S was established at Princess Margaret Cancer Centre (Canada), and all other cell lines were obtained from American Type Culture Collection (ATCC). Metformin (1,1-Dimethylbiguanide hydrochloride) was purchased from Sigma Aldrich.

### Proliferation assays

Cells were seeded into black, clear-bottom 96-well assay plates and incubated for 24 hours, prior to treatment with serially increasing concentrations of metformin ranging from 1–50mM. After 72 hours exposure to metformin, alamarBlue^®^ (Life Technologies) reagent was added and incubated for 4 hours. Fluorescence intensity was measured, and inhibition of proliferation was calculated by normalizing to an untreated control.

### Oxygen consumption rates

Cells were seeded into 96-well plates and incubated for 24 hours prior to measuring the oxygen consumption rate (OCR) with the Seahorse XF96 Extracellular Flux Analyzer (Seahorse Bioscience). Basal OCR was measured in untreated cells for 30 min prior to injection of metformin into the wells yielding final concentrations of 0.02mM, 0.5mM, 2mM, and 10mM. OCR was then measured for up to 8 hours. Inhibition of OCR was measured by normalizing to the basal OCR, and comparing to an untreated control.

### RNA extraction and qPCR

RNA was isolated from cells with TRIzol reagent and reverse transcribed into cDNA. Subsequently, qPCR was performed to measure expression of OCT1, OCT2, OCT3, MATE1 and MATE2 using SYBR green. All experiments were performed in triplicate and repeated independently 3 times. Our aim was to compare the relative gene expression of metformin transporters across cancer cell lines of various origins. Due to individual differences as well as the frequent genetic alterations in cancer, we reasoned that a normalization factor derived from multiple reference genes would be most robust. We first selected 12 candidate reference genes based on a study by Popovici et al. who compared 10 public microarray datasets to identify potential stable housekeeping genes between patients with different cancer types [52]. We cross referenced these candidate genes to our microarray data from different cell lines [53–55] and eliminated 8 that were regulated in response to hypoxia. This left the 4 genes HPRT1, HSP90AB1, RPL13A and YWHAZ as reference genes. To derive a normalization factor, we first measured the relative expression of these 4 genes and normalized the expression of each to be equal to 1 across all samples. This was done to prevent more highly expressed genes from contributing more to the normalization factor. Then, we eliminated the highest and lowest expressed gene for each sample, in order to prevent skewing of results by the inclusion of aberrantly expressed genes due to events such as genetic amplification or deletion, which commonly occur in cancer. The normalization factor for each sample was calculated as the average expression of the 2 remaining genes. Primers are listed in S1 Table.

### Metformin concentration by HPLC

Cells were treated with 2 mM metformin in media for 30 minutes and washed 3 times. Cells were harvested, lysed and mechanically homogenized in water. Homogenate was mixed with 100 ng of D^6^-Metformin. Proteins were precipitated from the homogenate with acetonitrile and reconstituted in 50% methanol. Metformin was quantified by HPLC coupled with a mass spectrometer (Applied Biosystems, Foster City, CA, USA). To determine the average volume of cells, each cell line was analyzed with the Vi-Cell XR (Beckman Coulter) according to manufacturer’s instructions. Intracellular metformin content was normalized to either cell number or cell volume.

### Xenografts and tumor hypoxia

All animal experiments were performed under protocols approved by the Ontario Cancer Institute’s (Princess Margaret Cancer Centre) Animal Care Committee. 1x10^6^ A549 cells in a 1:1 matrigel:media solution were injected in the right flank of Non-Obese Diabetic- Severe-Combined Immune-Deficient (NOD-SCID) mice. Mice were anesthetized for cell injection by isoflurane. Tumor volume was determined 2 times per week by caliper measurement using the formula Volume = Length·(Width^2^)·0.5. When xenografts reached a volume of 1,000 mm^3^, mice were given 0–10mg/ml metformin in the drinking water. One week later, they were injected intraperitoneally with 30 mg/kg EF5 (2-(2-Nitro-1 H- imidazole-1-yl)-N-(2,2,3,3,3-pentafluoropropyl)acetamide), a kind gift from Dr. C. Koch (University of Pennsylvania, Philadelphia, PA, USA). After three hours, mice were sacrificed by cervical dislocation under anesthesia. Blood was collected by cardiac puncture. Tumors were excised, and a piece was frozen for metformin quantification by HPLC (see above). The rest of the tumor was submerged in Optimal Cutting Temperature Compound and immediately frozen in liquid nitrogen for immunohistochemistry. Tumor sections measuring 5 mm were stained with a Cy3-conjugated antibody recognizing EF5 (Dr. C. Koch, University of Pennsylvania), and DAPI (Sigma) for DNA. Slide images were scanned in on a TissueScope whole slide immunofluorescence scanner (Huron Technologies, Waterloo ON), then stained for Hematoxylin and Eosin, to identify necrotic from viable tumor tissue, and re-scanned. Scans were aligned with ImagePro Plus software (Media Cybernetics, Rockville MD) and analyzed with Definiens TissueStudio suite (Definiens AG, Munich Germany). Regions of interest denoting viable tumor were separated from surrounding normal tissue, and from necrotic regions within the tumor, using the morphological information in the Hematoxylin and Eosin channels via a machine learning classifier. Following a quality assurance check, a threshold for positive EF5 signal was set in relation to mean background signal by reviewing multiple images, and the number of EF5 positive cells were counted, and quantified as a percentage of the cells present in the viable tumor area.

### Statistical analysis

For proliferation AUC calculations, metformin concentration was log10 transformed and proliferation normalized to control. OCR AUC was first derived as a function of time for each individual metformin concentration and then calculated after transforming the metformin concentrations log 10. For marker associations, Pearson’s and Spearman’s coefficient *r* were used to measure correlations between the tested variables. For metformin concentrations in cells, tumors or blood, as well as for hypoxic fractions, Student’s t-test was used. Correlations were deemed significant when *p*<0.05. Statistical testing was performed using GraphPad Prism software.

## Results

We first established a panel of cancer cell lines expected to capture potential heterogeneity in metformin response and OCT/MATE expression. Included in the panel were the cell lines A549 (lung adenocarcinoma) and BxPC-3 (pancreatic adenocarcinoma), which have been previously shown to be particularly sensitive to the growth-inhibitory effects of metformin because of mutations in LKB1 (STK11)(A549) or mitochondrial complex I (BxPC3) [56, 57]. We included HeLa (cervical carcinoma) as another cell line carrying an LKB1 mutation, and HCC827 (glioma) and U937 (lymphoma) as other cell lines with mutations in complex I [56]. LNCaP (prostate adenocarcinoma) was included since we have previously shown that these cells are sensitive to the inhibition of respiration by metformin [10]. We also included HCT116 and POP-092S (colorectal adenocarcinoma) because we have previously shown that metformin can reduce hypoxia in xenografts established from these cells [10]. Using publicly available microarray data [58], we further identified cell lines with high expression of OCTs or MATEs in order to explore potential correlations with metformin sensitivity. This resulted in inclusion of RCC4 (renal carcinoma), HepG2 (hepatocellular carcinoma), FaDu (upper aerodigestive carcinoma) and T98G (glioma). Finally, we included more cell lines from cervix (ME180) and head and neck (SCC-16A/B, SCC-19A/B, SCC-74A/B) tumors since hypoxia is known to represent a strong negative prognostic factor in these sites [28, 59], rendering them relevant for translation of metformin as a hypoxic modifier. The head and neck cancer (HNC) cell lines represented matched pairs originating from the same patient, where A was established from the primary tumor and B from the metastatic site. This resulted in a panel of 19 different cell lines ranging in expected metformin sensitivity and transporter expression.

**Fig 1:**
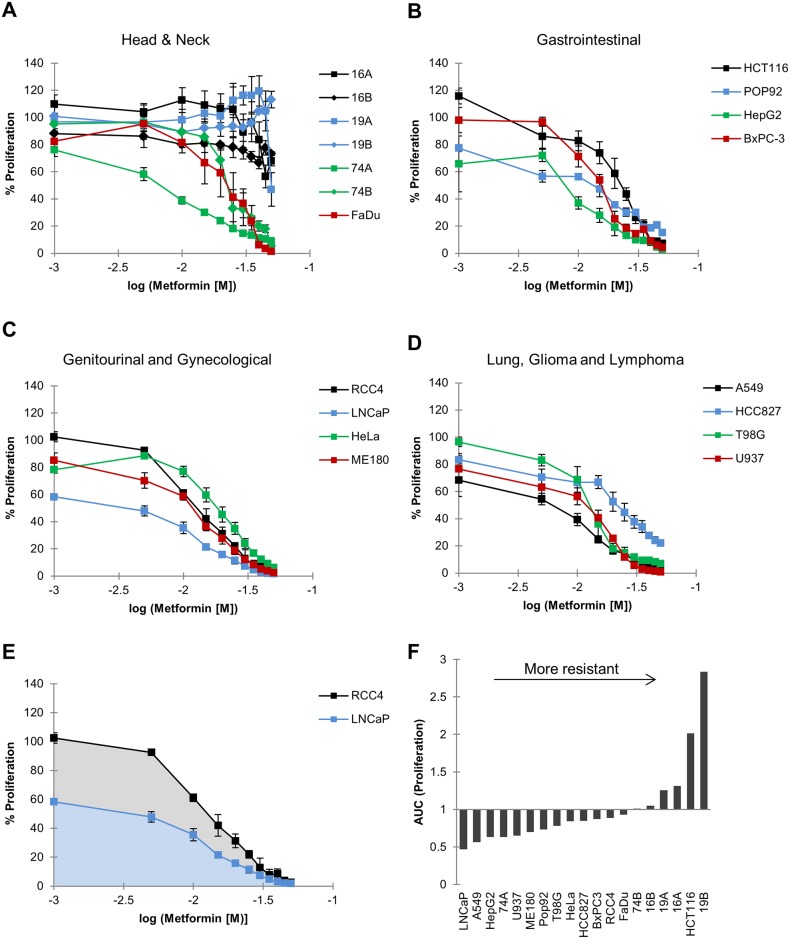
Metformin inhibits proliferation in a dose-dependent manner. A-D: Proliferation was quantified using an alamarBlue^®^ assay following 72 hours of metformin exposure and plotted against the log concentration of metformin. Values shown are expressed as a percentage of an untreated control. Mean ± S.E.M., *n* = 3. E: Examples of area-under-the-curve (AUC) values calculated from dose-response curves in A-D. Lower AUCs indicate greater sensitivity to inhibition of proliferation (LNCaP), while higher AUCs indicate resistance (RCC4). F: The AUC was normalized across the cell line panel and is shown after arranging cell lines in order of increased resistance.

We subsequently profiled the ability of metformin to inhibit proliferation across the panel using the alamarBlue^®^ assay. Data are presented in Fig 1, where cell lines are grouped by site of origin for clarity. Metformin inhibited proliferation in a dose-dependent manner in most of the cell lines in the panel (Fig 1A–1D). Notably however, HNC cell lines from patient 16 and 19 were extremely resistant to the anti-proliferative effect of metformin (Fig 1A). Also, the genetic evolution that had taken place in cells isolated from the primary site (A) versus the metastasis (B) of patient 74 resulted in resistance to metformin (Fig 1A). In order to compare metformin response quantitatively, we used the area under dose-response curve (AUC) as illustrated in Fig 1E. This metric does not require fitting to sigmoidal curves and can capture differences in dose-dependent responses that cannot be represented by IC_50_ statistics [60]. For the 15 cell lines where IC50 was reached, this correlated highly with AUC (r = 0.95) (S1 Fig). There was a clear tendency for HNC cell lines to be resistant to the anti-proliferative effects of metformin (Fig 1F). The cell line in our panel that was most sensitive to anti-proliferative effect of metformin was LNCaP. Consistent with previous reports [57], our data also identified the lung carcinoma line A549 as being sensitive to metformin (Fig 1F). Overall, these data suggest that substantial heterogeneity exists on a cell-autonomous level regarding the sensitivity of cancer cells to the anti-proliferative effects of metformin, emphasizing the need for appropriate predictive biomarkers.

**Fig 2:**
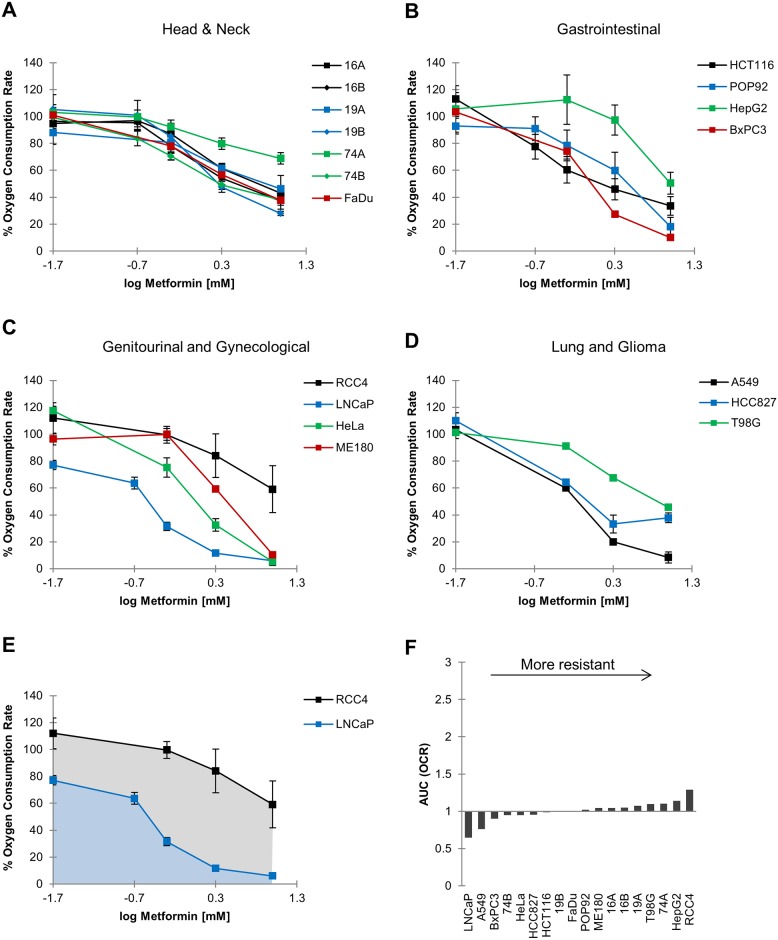
Metformin inhibits oxygen consumption in a dose-dependent manner. A-D: Oxygen consumption rate (OCR) was quantified using the Seahorse Bioanalyzer following 8 hours of metformin exposure and plotted against the log concentration of metformin. Values shown are expressed as a percentage of an untreated control. Mean ± S.E.M., *n* = 3. E: Examples of area-under-the-curve (AUC) values calculated from dose-response curves in A-D. Lower AUCs indicate greater sensitivity to inhibition of oxygen consumption, while higher AUCs indicate resistance. F: The AUC was normalized across the cell line panel and is shown after arranging cell lines in order of increased resistance.

Next, we assessed the ability of metformin to inhibit cellular oxygen consumption in our cell lines. Using the Seahorse Extracellular Flux Analyzer, we measured oxygen consumption rates (OCR) of cells for up to 8 hours following treatment with 0.02–10 mM metformin. Metformin consistently inhibited OCR in a dose- and time-dependent manner (Fig 2A–2D), also in the HNC cell lines that were resistant to the anti-proliferative activity (Fig 2A). To quantify individual cellular sensitivity to OCR inhibition, we derived the AUC of the dose-response following 8h of metformin treatment similar to above (Fig 2E). This revealed that there was a substantially more uniform response across cell lines to the inhibition of oxygen consumption by metformin (Fig 2F) compared to the inhibition of proliferation (Fig 1F). Similar to the inhibition of proliferation, LNCaP and A549 cells were most sensitive to the inhibition of oxygen consumption by metformin (Fig 2F). It is interesting to note that we had previously measured a drop in OCR and hypoxic fraction *in vivo* in tumors derived from cell lines of average sensitivity (HCT116 and POP92) within this panel. This suggests that most tumors (or xenografts) are susceptible to inhibition of oxygen consumption by metformin and thus benefit from its use as a hypoxia modifier.

There was no association between the inhibition of OCR and proliferation across the panel (S2 Fig). This suggests that differences in signaling pathways downstream of mitochondrial inhibition determine the ultimate anti-proliferative response.

**Fig 3:**
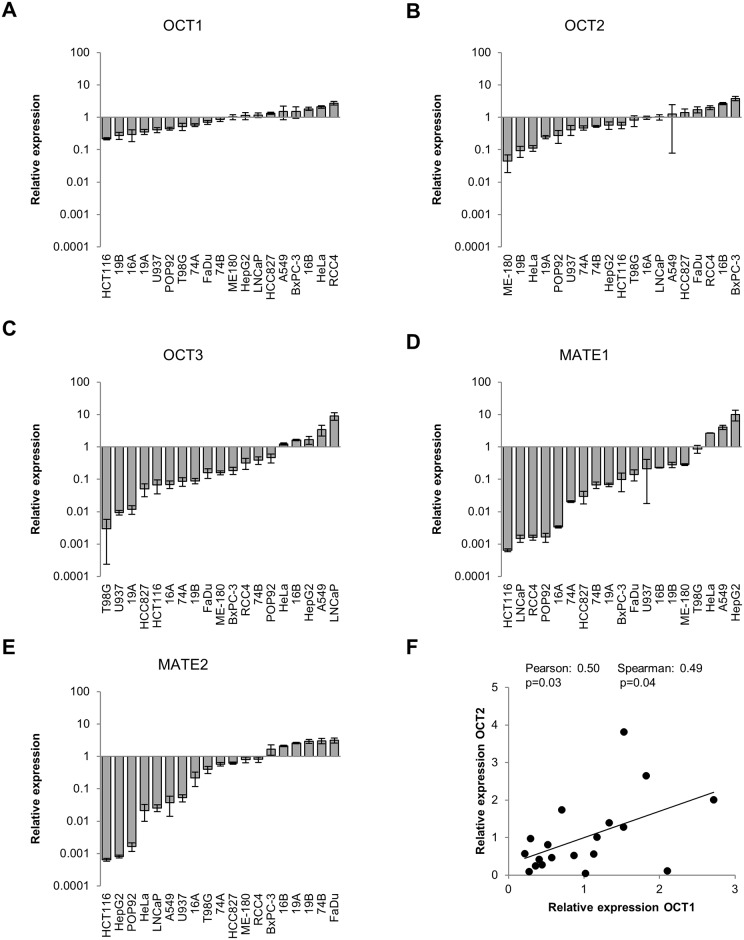
Expression of OCTs and MATEs across cell lines. A-E: Relative mRNA expression of OCTs and MATEs was normalized across the cell line panel and is shown after arranging cell lines in order of increased expression. Mean ± S.E.M., *n* = 3. F-G: Relative expression of OCT1 correlated significantly with OCT2 (F) between cell lines.

We next sought to quantify OCT and MATE expression in our panel of cell lines, using real-time PCR. We chose to examine this on mRNA level given that quality antibodies did not exist for all transporters. Our data show that there is relatively similar expression of OCT1 across cell lines (Fig 3A) (variance: Var = 0.49). In comparison, there is higher variability in OCT2 expression (Fig 3B) (Var = 0.96). Interestingly, OCT3 showed the highest overall expression of the OCT transporters, and also most substantial variability (Var = 4.5). MATE1 and MATE2 expression also varied considerably among our cell lines (Fig 3D and 3E) (Var = 5.83 and 1.37 respectively). These general results are in line with published microarray data, which also indicate low and uniform OCT1 and OCT2 expression, and high and variable expression of OCT3, MATE1 and MATE2 across cancer cell lines [58]. Furthermore, we observed a significant correlation between OCT1 and OCT2 expression (Fig 3F), and between OCT1 and OCT3 expression (S3 Fig). The latter was only statistically significant when considering the Spearman correlation, due to one outlier data point. The statistical association between the expression of different OCTs may originate from alteration (methylation/amplification/deletion) of the common locus in the unstable cancer genome, but the relevance of this remains unclear. No relationship was found between the expression of other markers (data not shown).

**Fig 4:**
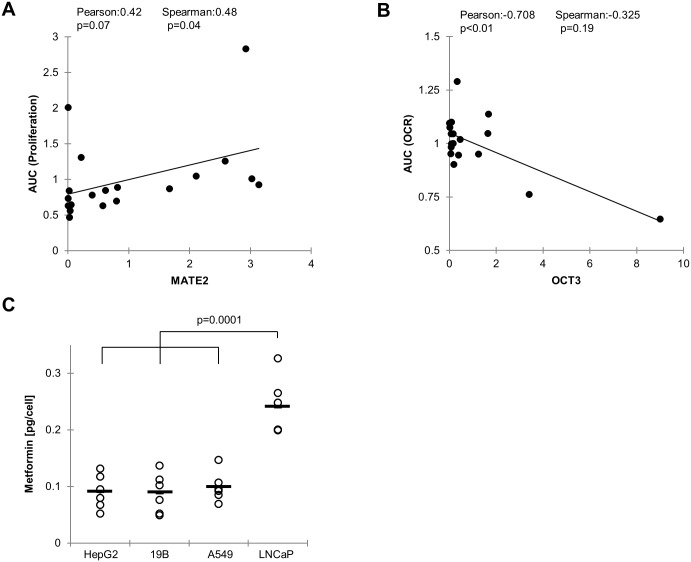
Metformin response is associated with MATE2 and OCT3 expression. A-B: AUCs for proliferation inhibition (A) and OCR inhibition (B) were correlated with MATE2 and OCT3 expression respectively. C: Intracellular metformin concentration after 30 minutes exposure to 2mM metformin as measured by HPLC. Circles represent biological replicates, the mean value is indicated with a bar. Assuming a cell volume of 2pL, 1mM corresponds to 2.5pg/cell.

As transporter-mediated uptake and retention of metformin would be expected to contribute to the ability of the drug to inhibit mitochondrial respiration and proliferation, we sought to determine whether OCT and/or MATE expression correlate with response. We therefore analyzed associations between OCT and MATE expression and inhibition of proliferation or OCR by correlating individual transporter expression with AUCs derived for each cell line. We observed that MATE2 expression correlated with resistance to the anti-proliferative activity of metformin (Fig 4A). Furthermore, OCT3 expression correlated with sensitivity to inhibition of OCR (Fig 4B). These correlations were in the expected directions given the known roles of OCT3 and MATE2 as metformin importer and exporter respectively. The significant correlation between OCT3 and OCR inhibition was largely driven by the prostate cancer cell line LNCaP and not significant when considering the Spearman rather than Pearson correlation (Fig 4B).

LNCaP clearly stood out in the panel since it was the most sensitive to metformin in regards to inhibition of both OCR and proliferation, and it had high OCT3 expression combined with low expression of both MATE1 and MATE2. Other cell lines with high OCT3 expression, such as A549 and HepG2, also had high expression of either MATE1 or 2, which would be expected to counteract intracellular metformin accumulation. Consistent with this, we found that LNCaP cells accumulated significantly higher concentrations of metformin compared to A549, HepG2 or 19B cells (Fig 4C).

**Fig 5:**
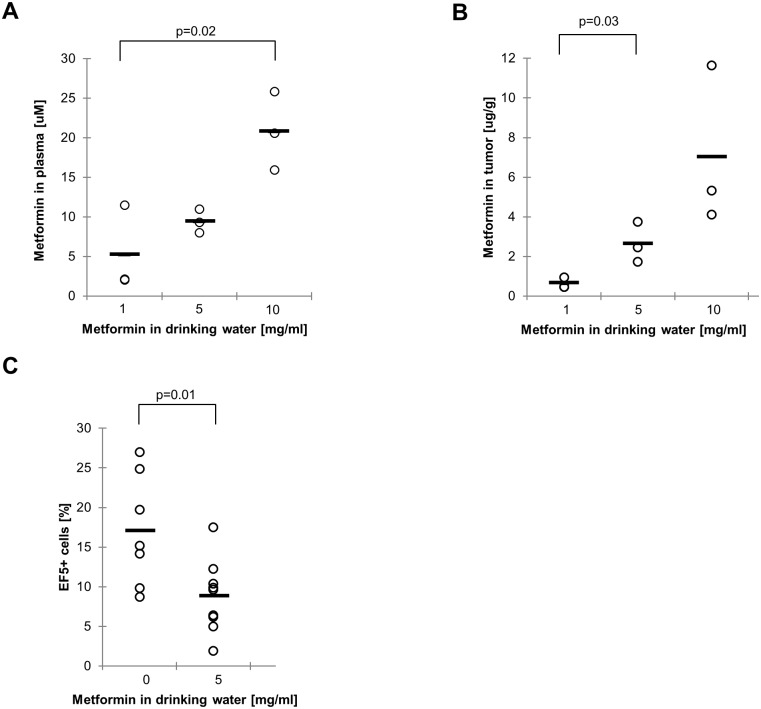
Metformin reduces tumor hypoxia. Mice bearing subcutaneous A549 xenografts were given metformin in the drinking water to the indicated concentration. A-B: After 1 week, metformin concentrations in plasma (A) and tumor (B) were measured using HPLC. Assuming the density of tumor tissue is 1g/ml, 1ug/g corresponds to ~8uM. C: After 1 day, mice were administered EF5 to label hypoxic tumor cells. Viable EF5-positive cells were quantified from tumor sections. Circles represent the average value from two sections of a tumor; the mean value across all tumors is indicated with a bar.

In diabetic patients, the average plasma level of metformin is 20 μM [61]. In the present study, only the most sensitive cell line (LNCaP) demonstrated a statistically significant acute reduction in oxygen consumption in response to this dose. However, metformin concentrations could accumulate in tissues over time, and tumor cells may be more sensitive in the tumor microenvironment which is less rich in nutrients and growth factors. Therefore, we tested if a clinically relevant metformin dose could influence oxygen consumption and reduce tumor hypoxia *in vivo*. First we established the concentration of metformin needed in the drinking water of mice to obtain a clinically relevant plasma concentration. 5 or 10mg/ml metformin in drinking water resulted in clinically relevant plasma concentrations of 9.5 or 20.8 μM respectively in the mice after one week (Fig 5A). The corresponding tumor concentrations were about twice as high (20–54 μM) (Fig 5B). We tested if the lower dose of 5mg/ml metformin could reduce hypoxia in A549 xenografts. To this end, we administered the hypoxic cell marker EF5 to tumor-bearing mice and quantified the fraction of EF5 positive tumor cells using immunofluorescence from tumor sections. Interestingly, metformin resulted in a significant decrease of the hypoxic tumor fraction after 1 day of metformin exposure (Fig 5C). However, this effect was transient as the hypoxic fraction was restored after 5 days exposure (data not shown). Taken together, these data suggest that tumors may accumulate higher concentrations of metformin than the steady-state plasma levels, and that moderate clinical doses can result in reduced tumor hypoxia.

## Discussion

In this study, we have documented relationships between the anti-proliferative and anti-respiratory effects of metformin *in vitro* in a panel of 19 cancer cell lines, and correlated this with expression of known metformin transporters. One important finding is that a subset of cell lines is very resistant to the anti-proliferative effects of metformin, justifying the existence and importance of predictive biomarkers. This subset appeared to be enriched for cell lines derived from head and neck cancer (16A/B, 19A/B, 74B and FaDu). Interestingly, expression of MATE2 correlated with resistance to the anti-proliferative effect of metformin in the panel (r = 0.5, p = 0.04). This correlation was even stronger when two single outlier replicates were excluded from analysis (r = 0.6, p<0.005)(data not shown). MATE2 expression was not associated with the more short-term endpoint of respiration inhibition. This may possibly be due to the function of MATEs as drug/H+ antiporters, rendering them more efficient over time as the extracellular pH drops due to increased glycolysis and lactate secretion [62].

The statistical association between MATE2 and inhibition of proliferation was weak. This is not surprising given that many other additional factors exist that will influence both metformin concentration and response. Such factors may include other transporters not assessed here, as well as basal mitochondrial function and metabolic adaptation. Also, our study was limited to measuring mRNA expression of metformin transporters, whereas protein expression would be expected to correlate better with function. Our cell line panel was highly diverse, with cancer cell lines that have arisen from many different sites of origin through many different combinations of genetic alterations. In light of this, one might expect correlations to be stronger within a clinical disease site, between tumor cells that share certain somatic mutations [56], and potentially when using protein rather than mRNA expression. Our study does not rule out the existence of associations between OCTs, MATE1 and response in more homogeneous samples and/or on protein level. Nevertheless, the existence of a weak statistical association between MATE2 expression and the anti-proliferative effect of metformin suggests that tumor MATE2 expression should be evaluated as a potential biomarker of response in ongoing clinical trials that are investigating the effects of metformin on tumor (re)growth. Furthermore, it is unclear at this point to what extent cell autonomous effects drive the anti-cancer effects of metformin. If relationships between metformin response and transporter expression in patient tumors are identified, this will serve as a strong argument for a substantial cell-autonomous component.

In comparison to proliferation, there was much less variability between the responses of cell lines to the effects of metformin on oxygen consumption, and no resistant cell lines were identified. This is important given the implication that metformin potentially could be used universally in the context of combination with radiotherapy to reduce the hypoxic tumor fraction. Our data hence suggest that all cancer cell lines express sufficient levels of OCT family members to take up metformin and affect mitochondrial physiology. Taken together, our data also suggest that uptake is not a limiting factor for the anti-proliferative effects of metformin, which instead is governed by genetic variation in signaling pathways, individual vulnerabilities and efflux driven by MATE2 expression.

Although our data suggest that all cell lines are sensitive to the mitochondrial effects of metformin, a weak correlation was observed between inhibition of oxygen consumption and OCT3 expression. This however, was largely driven by one data point represented by LNCaP which has an unusually high OCT/MATE ratio. Our data are consistent with a model where extremely high OCT3 expression (Fig 3C) results in high intracellular metformin concentration (Fig 4C), and extreme response in terms of inhibition of oxygen consumption (Fig 2F), proliferation (Fig 1F) and reduction of tumor hypoxia [10]. However, this condition may be rare (as observed in our cell line panel) and the general correlation would have to be corroborated in a much larger panel that includes other cell lines with OCT/MATE ratio of similar magnitude.

In this study, we established a treatment protocol that resulted in long-term stable metformin exposure closely resembling that of diabetic patients. Using this schedule, we found that metformin accumulated in the A549 tumors to about double of the plasma concentration after 1 week of metformin administration. Another recent study found plasma and tumor concentrations of metformin to be similar after 1 week exposure, using a HCT116 tumor model [63]. It will be interesting to assess whether this relationship holds in orthotopic experimental tumor models and in human tumors *in situ*, where the tumor microenvironment and drug delivery may differ. In a vast majority of cell lines *in vitro*, proliferation and oxygen consumption were only affected by metformin at concentrations substantially higher than those achieved in plasma or tumors. However, as pointed out in a recent report by Chandel et al., sensitivity to metformin is known to be higher when nutrients are limited, like in the tumor microenvironment [63]. It is therefore speculated that lower concentrations of metformin are required *in vivo* for anti-neoplastic activity [63]. In line with this hypothesis, a recently published paper demonstrated that the amounts of metformin achieving similar levels of AMPK activation in xenografts compared to tissue culture differed by a factor of 300 [64]. Several clinical studies have also demonstrated that anti-diabetic doses cause decreased tumor cell proliferation, and here we show that similar doses causes reduced hypoxia in xenografts. Taken together, these data strongly argue that anti-diabetic doses of metformin affect tumor physiology in different ways that can be taken advantage of clinically.

It remains unclear why the effect of metformin on the hypoxic tumor fraction was transient in this study. The magnitude of the zone of hypoxic cells situated between well-oxygenated cells and necrotic areas is a result of oxygen delivery, consumption and cell viability (tolerance). It is possible that cellular hypoxia tolerance is the ultimate determinant of steady states of hypoxia, and that changes in supply and demand can only have transient effects. The response kinetics are likely different in patients and mice, making it hard to predict optimal scheduling in patients. The longitudinal monitoring of tumor microenvironment in cancer patients given metformin will be a challenging but important task going forward.

## Supporting Information

S1 Fig AUC and IC50 are highly correlated. IC50 values could be calculated for 15 out of 19 cell lines and correlated with AUC. Correlation coefficient: ƍ = 0.95.
(TIF)

S2 Fig The anti-respiratory and anti-proliferative effects of metformin are not correlated. AUCs for proliferation inhibition and OCR inhibition were correlated, no statistically significant association was found.
(TIF)

S3 Fig Relative expression of OCT1 correlated significantly with OCT3 between cell lines. (TIF)

S1 File Data. File containing data for oxygen cunsuption rates (OCR), proliferation and expression of OCT1, OCT2, OCT3, MATE1 and MATE2 across cell lines.
(XLSX)

S1 Table Primer pairs for qPCR. (TIF)
