# Supplementary figures and images for: MATE2 Expression Is Associated with Cancer Cell Response to Metformin
Source: PLoS One. 2016 Dec 13;11(12):e0165214. doi: 10.1371/journal.pone.0165214 (PMC5154501; doi:10.1371/journal.pone.0165214)

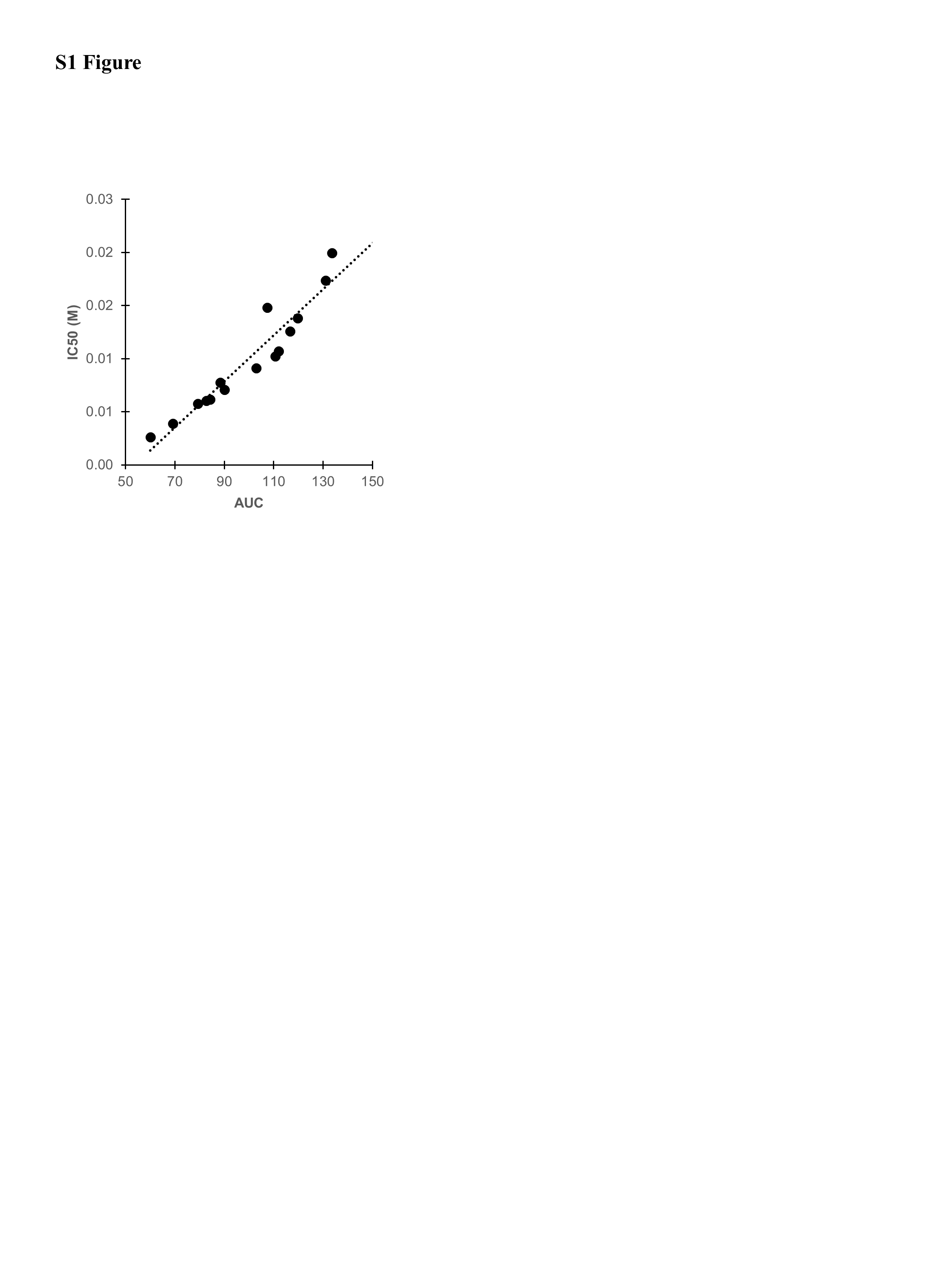

Supplement: S1 Fig — IC50 values could be calculated for 15 out of 19 cell lines and correlated with AUC. Correlation coefficient: ƍ = 0.95. (TIF) [file pone.0165214.s001.tif]

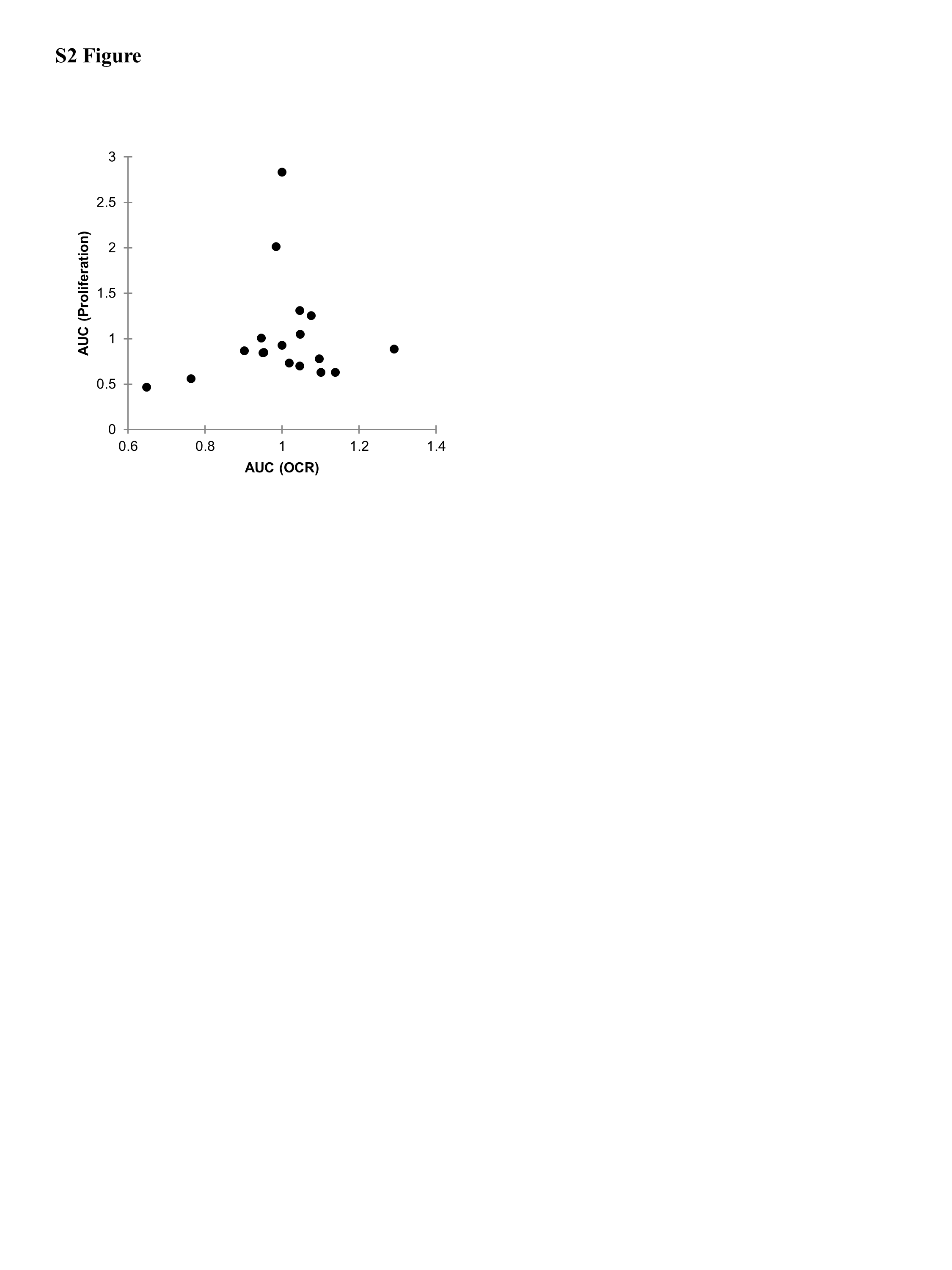

Supplement: S2 Fig — AUCs for proliferation inhibition and OCR inhibition were correlated, no statistically significant association was found. (TIF) [file pone.0165214.s002.tif]

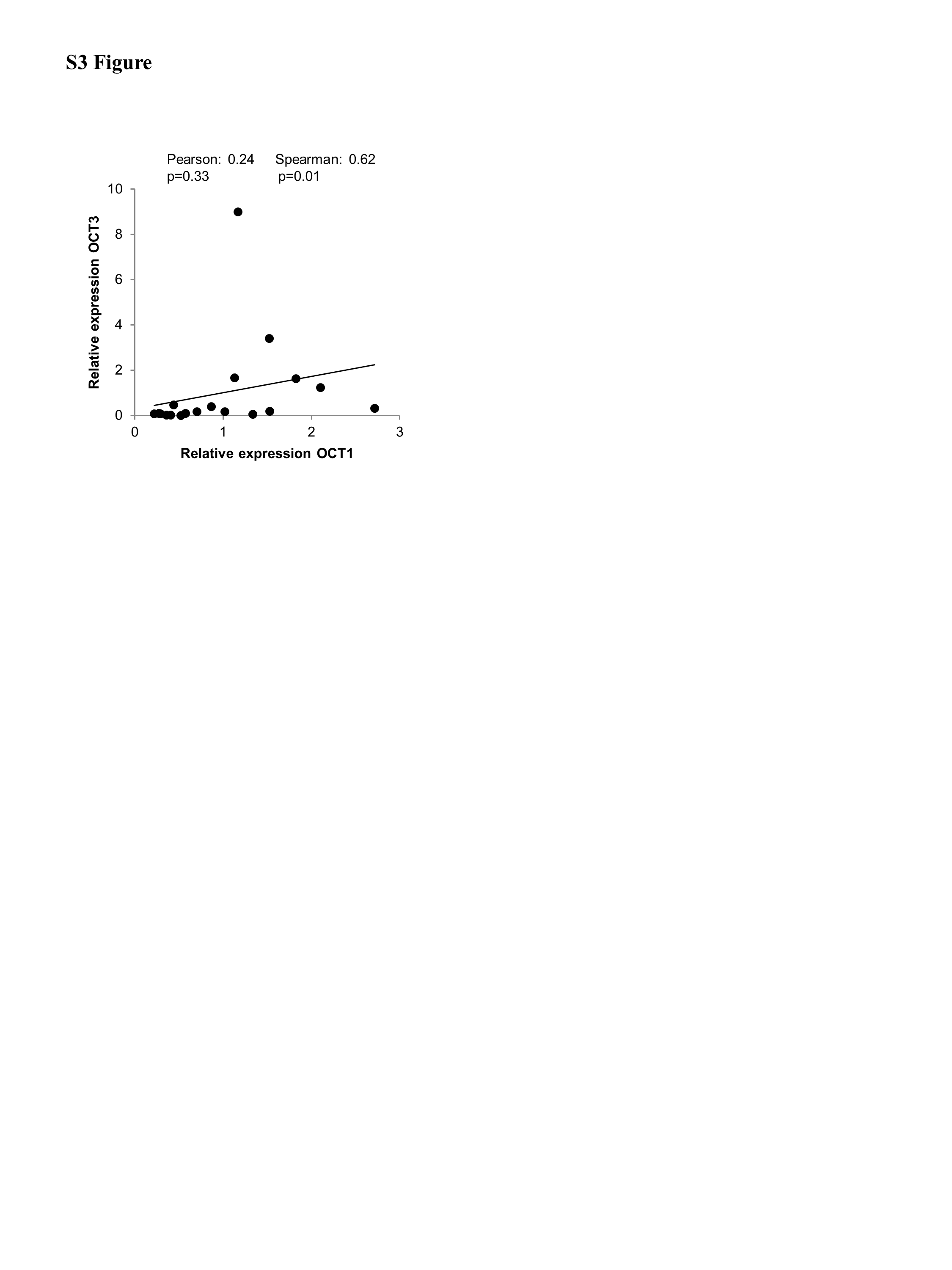

Supplement: S3 Fig — (TIF) [file pone.0165214.s003.tif]

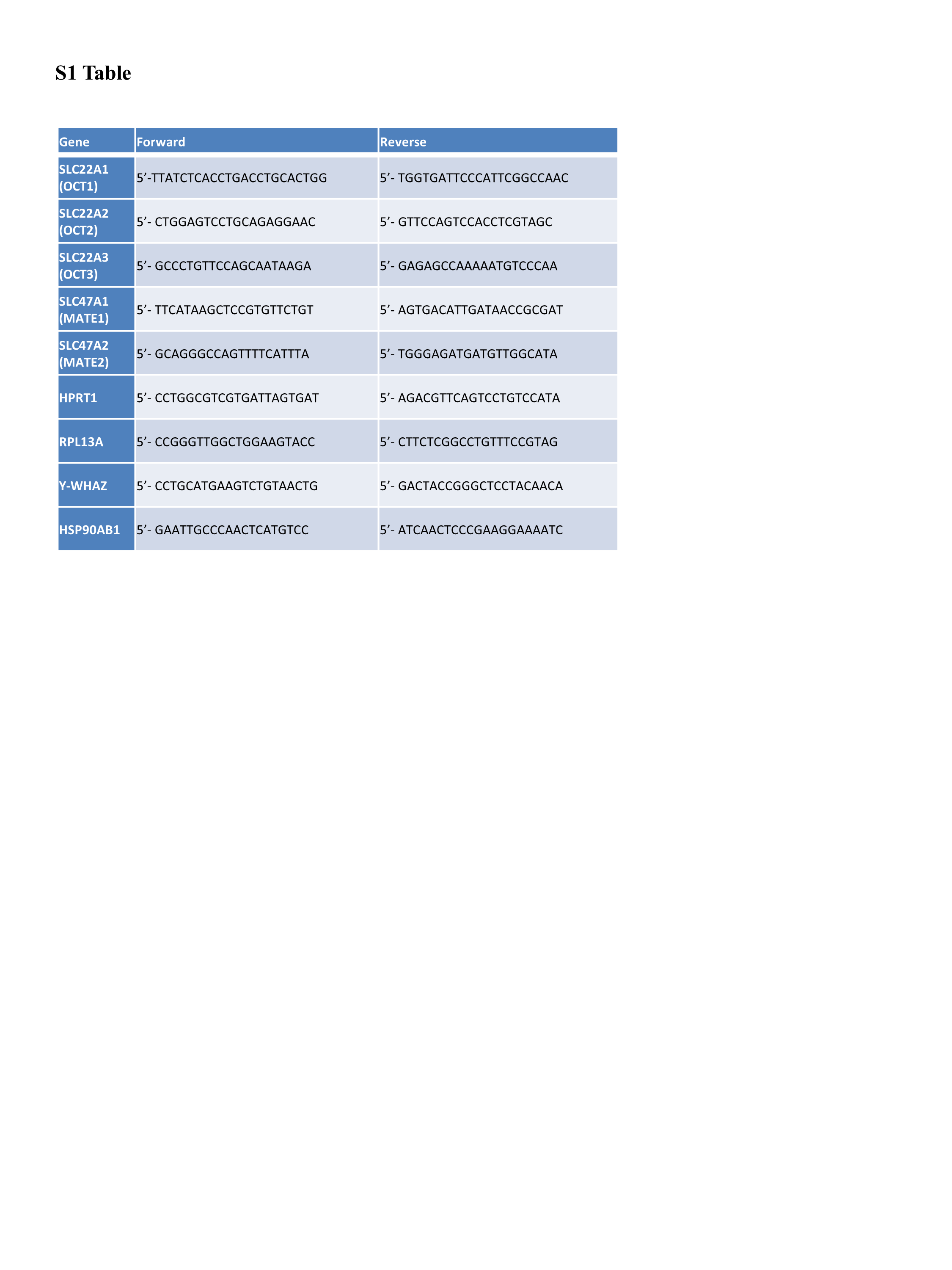

Supplement: S1 Table — (TIF) [file pone.0165214.s005.tif]
